# Trends and disparities in tuberculosis burden in Kazakhstan and Mongolia (2017–2021): a comparative analysis using GBD metrics

**DOI:** 10.3389/fpubh.2025.1575107

**Published:** 2025-05-15

**Authors:** Oyunzul Amartsengel, Malika Idayat, Alexander Rommel, Natalya Glushkova, Kairat Davletov, Malik Adenov, Naranzul Dambaa, Lkhagvasuren Khorolsuren, Elena von der Lippe

**Affiliations:** ^1^Department of Health Policy, Mongolian National University of Medical Sciences, Ulaanbaatar, Mongolia; ^2^Department of Medicine and public health, Al-Farabi Kazakh National University, Almaty, Kazakhstan; ^3^Robert Koch Institute, Berlin, Germany; ^4^Science and Technology Park, Asfendiyarov Kazakh National Medical University, Almaty, Kazakhstan; ^5^Research Institute of Phthisiopulmonology, Almaty, Kazakhstan; ^6^National Center for Communicable Disease, Ulaanbaatar, Mongolia

**Keywords:** disability-adjusted life years, years of life lost, years lived with disability, TB, Central Asia

## Abstract

**Background:**

In Central Asia, respiratory diseases, particularly tuberculosis (TB), are widespread communicable diseases that significantly impact both individuals and health systems, posing a substantial burden. Research highlights the importance of assessing the impact of TB on global morbidity statistics, given its status as a prominent contributor to global morbidity rates and the cause of over a million deaths annually. Our study aims to assess the patterns and changes in the burden of TB in Mongolia and Kazakhstan.

**Methods:**

The design is retrospective cross-sectional study. This study used the Global Burden of Disease (GBD) framework, which introduced disability-adjusted life years (DALY) as a measure of disease burden, combining mortality (Years of Life Lost, YLL) and morbidity (Years Lived with Disability, YLD). The calculations were based on standard GBD formulas, incorporating life expectancy data, age at death, and disease-specific disability weights. We calculated Mongolia’s and Kazakhstan’s national TB registration data from 2017 to 2021. From 2017 to 2021, Kazakhstan and Mongolia experienced significant declines in the burden of TB, as indicated by reductions in years of life lost and years living with disability.

**Findings:**

From 2017 to 2021, Kazakhstan’s YLL decreased by 18.2% and YLD by 36%, reflecting improved TB control. Mongolia experienced a 24.9% decline in YLL and a 39.4% reduction in YLD, although premature mortality in older men remains a challenge. These findings highlight the need for targeted interventions and healthcare equity to sustain TB control efforts. YLD rates remained low and had minimal impact on total DALYs, underlining the positive trends in reducing TB mortality and disability in both countries. Kazakhstan and Mongolia have significantly reduced the burden of TB, evidenced by reductions in DALY, YLL, and YLD rates.

**Interpretation:**

The results suggest that while TB control efforts have yielded positive results in both countries, Mongolia faces challenges in reducing TB-related mortality and morbidity, highlighting the need for targeted interventions and improved access to TB services. These results are consistent with global trends showing a declining TB burden due to improved diagnostics and treatment strategies but highlighting structural disparities that hinder uniform progress. Moreover, WHO estimates for the Western Pacific Region (WPR) and South-East Asia Region (SEAR) reported a slower increase in tuberculosis mortality trends from 2017 to 2021. Future research should focus on addressing the factors contributing to Mongolia’s persistently high TB burden, including access to healthcare, treatment adherence, and the role of comorbidities. In addition, expanding the scope of analysis to other Central Asian countries will provide a broader understanding of TB control efforts across the region.

## 1 Introduction

Tuberculosis (TB) has probably returned to being the world’s leading cause of death from a single infectious agent, following 3 years in which it was replaced by coronavirus disease (COVID-19) (1). Thus far, controlling the transmission of TB remains one of the most critical global health challenges. The “burden of TB” concept is a valuable framework for comprehensively evaluating the epidemiological landscape, using data on TB incidence and prevalence (2). TB is a significant contributor to the overall global burden of disease, resulting in a staggering number of over 1.6 million deaths each year (3).

The majority of people registered with TB (about 90%) are adults, and the disease is more prevalent in men than in women (4). TB is a curable disease, if diagnosed timely and treated effectively. Without treatment, the mortality rate (MR) is high.

Standard treatment for drug-susceptible TB usually lasts 6 months, and for Rifampicin/multidrug-resistant TB, up to 18 months (3). Besides the duration, TB treatment requires a lot of effort from the service providers (e.g., management of side effects) as well as from the patients (treatment adherence) to ensure continuation of treatment, and to avoid (further) development of drug-resistant TB.

During the COVID-19 pandemic, in 2020 and 2021, TB incidence rates decreased in high-prevalence countries, but MR increased. This was due to the lack of diagnosis and treatment (5). In 2021, the estimated global TB case numbers reached approximately 10.6 million cases (95% uncertainty interval [UI]: 9.9–11 million), corresponding to an incidence rate of 134 cases per 100,000 population (95% UI: 125–143). Overall, 6.7% of TB cases worldwide occur in individuals who are living with HIV. Geographically, most TB cases in 2021 were concentrated in a few regions defined by the World Health Organization (WHO). South-East Asian Region constituted the highest proportion with an incidence of 234 per 100,000, followed by the African Region with 212 per 100,000 and the Western Pacific Region with 98 per 100,000 population. Comparatively lower proportions were observed in Region of Americas (30 per 100,000) and the European Region (25 per 100,000) (5).

In Central Asia, respiratory diseases, particularly TB, are prevalent and impose a significant burden on individuals and health systems (6). Except during the COVID-19 pandemic, TB helds the remarkable distinction of being the leading cause of death attributed to a single infectious agent (3).

Considering the paramount focus on equitable access to TB diagnosis and treatment in the context of global health goals, it is of utmost importance to also assess the gender differences in the burden of TB (7). TB incidence rates are notably higher in men, especially in developed countries, and in many cases, due to different social contact patterns, men tent to contribute more to ongoing transmission in the community than women (8). Therefore, addressing the burden of disease and potential gender differences in access to TB prevention, diagnosis and care is of paramount importance not only for men’s health but also for the overall prevention and treatment of TB (9).

According to the WHO Global TB Report 2024, the progress made in reducing tuberculosis (TB) mortality between 2015 and 2023 fell short of the target set in the “End TB Strategy.” The observed reduction of 23% is considerably below the desired goal of a 75% reduction by 2025 (1). Since 2012, there has been a notable upward trend in newly diagnosed TB cases in many countries (10).

Central Asia remains a major hotspot for drug-resistant TB (DR-TB), with outdated and costly treatment services facing financial constraints. Stigma and discrimination further exacerbate the issue. Following WHO guidance, Kazakhstan and Mongolia adopted the DOTS program in 1998 (11) and 1994, respectively (12). In recent years, local health initiatives have been implemented to enhance the population’s quality of life.

According to a study by Terlikbaeva et al., Kazakhstan is among those high-priority countries with TB incidence >20 cases per 100,000 population (13). In 2010, the TB incidence rate in Kazakhstan was 166 cases per 100,000 population. This rate was significantly higher than in 2015, as reported by Kyu et al. (14), which showed a decrease to 107 cases per 100,000 population. According to WHO estimates for 2023, the mortality rate from tuberculosis among HIV-negative individuals in Kazakhstan was projected to be 360 cases (range: 230–520), equating to 1.8 (range: 1.2–2.5) deaths per 100,000 population (15, 16).

Mongolia has the fourth highest number of TB cases among 37 countries in the Western Pacific Region of the WHO. Newly diagnosed case numbers have increased steadily since 1996 and decreased since 2007. WHO estimated the incidence of TB to be 428 per 100,000 population in 2021, while only 72 were officially registered. The detection of new cases has, therefore, decreased. 55.9% of registered cases were male and 41.1% female (17).

Kazakhstan and Mongolia provide compelling comparative approaches in the fight against TB. Despite differing healthcare systems and socioeconomic contexts, both nations face high TB incidence rates and significant infection control challenges. Each country is classified as a priority country for TB control by the WHO. Each has long implemented poverty reduction programs in line with WHO recommendations, including the directly observed treatment, short-course (DOTS) strategy. Although WHO actively promoted DOTS in the past, it is absent from the latest Global TB Report, likely due to advances in diagnostics, improved drug regimens for DR-TB, and evidence questioning its effectiveness in some contexts (1). Both countries face challenges stemming from underfunding, dependence on international grants, and difficulties in providing health services to remote and impoverished areas with elevated TB risk factors, such as poverty, migration, and limited health literacy. The funding gap in Mongolia for tuberculosis control, as outlined in the budgets included in the National Strategic Plans for Tuberculosis Control for 2024, was approximately 10% (equivalent to $1.6 million USD). Kazakhstan utilizes digital patient registries, enhancing the capacity for epidemiological studies, while Mongolia is focused on advancing its diagnostic and monitoring systems. A comparison between the countries illustrates how similar TB control measures work in different contexts, providing valuable insights for designing effective infection control strategies, diagnosis, and treatment in different regions.

Research highlights the importance of assessing the impact of TB on global morbidity statistics, given its status as a major contributor to global morbidity and the cause of over one million deaths annually^1^. Despite progress in TB control, there is limited comparative research analyzing DALY trends across Central Asian countries. Our study addresses this gap by using national data sources to assess the patterns and changes in the TB burden in Mongolia and Kazakhstan, with a particular focus on exploring gender disparities during the period 2017–2021. Furthermore, the observed trends and patterns are discussed in the context of health activities and policy measures introduced over the years to reduce mortality and improve TB treatment in both countries.

## 2 Materials and methods

### 2.1 Study design and data sources

The Mongolian data on TB were received from the Health Development Centre and the National Centre for Communicable Disease. We included all the registered cases between 2017 and 2021 on pulmonary and extrapulmonary TB nationwide. Mongolia has 32 TB dispensaries in 21 provinces, nine capital districts, prisons, and charity hospitals. The dispensaries register all diagnosed TB cases and send the data to the National Centre for Communicable Diseases (NCCD). The Tuberculosis Surveillance Centre of the NCCD collates the data and sends them to the Health Development Centre, which stores and processes all health data in Mongolia.

Data for Kazakhstan were sourced from the National Scientific Centre of Phthisiopulmonology (NSCP). The study encompasses all registered data for 2017-2021, including both pulmonary and extrapulmonary TB cases nationwide. In the Republic of Kazakhstan, all TB patients are meticulously registered in the National Tuberculosis Register, which operates under the purview of the Ministry of Health. The NSCP diligently collects and processes comprehensive health-related information about the population of Kazakhstan. TB cases are based on recorded data, with no further estimations or corrections.

In this study, we use the number of registered TB cases, all deaths due to TB, and the total population by age group and gender for Mongolia and Kazakhstan from 2017 to 2021. For prevalent cases in Kazakhstan, all TB cases were considered, including new cases, relapses, transfers, cases resuming treatment after a break, treatment failures, and other classifications. As only the case categories new and relapse cases are registered in Mongolia, we adjusted the calculations for Mongolia using the information available for Kazakhstan. The difference between prevalence and incidence was first calculated for each age group and applied as a multiplier to the incidence.

### 2.2 Indicators

To assess trends in TB epidemiology in both countries, we estimated several indicators of TB mortality and morbidity. Using the number of prevalent cases and the number of deaths, we assessed the mortality and case-fatality ratio as well as the years of life lost due to death (YLL) and disease (YLD) for the period between 2017 and 2021.

#### 2.2.1 Calculation of mortality and case fatality ratios

The mortality rate measures the occurrence of death in a defined population during a specified interval. The general formula for the mortality rate per 100,000 population over a given period is (18):


$$\text{Mortality rate}\;(\mathrm{MR})=\frac{\text{Number of deaths cases}\;}{\text{Population size for specified period}}\times 100\;000$$

We estimated the sex- and age-specific TB mortality rates for the whole observation period for both countries.

Case fatality rate is the ratio of deaths caused by a particular diagnosis to the total number of cases suffering from this cause. It expresses the lethal effect of a cause or disease. It is expressed as a percentage and is calculated as follows (19):


$$\begin{array}{l} \text{Case fatality ratio}\;(\mathrm{CFR},\text{in}\%)=\\ \frac{\text{Number of deaths cases}\;\mathrm{due}\;\text{to particular disease}}{\text{Total number of cases}\;\mathrm{due}\;\text{to same disease}}\times 100 \end{array}$$

Similarly, regarding the mortality rates, we estimated the case fatality rate due to TB for each sex and age group in both countries during the observed period.

### 2.3 DALY calculation

The Global Burden of Disease study (GBD), first conducted in 1990, introduced a new indicator for measuring the burden of disease: disability-adjusted life years (DALY) (20). Disability-adjusted life years (DALYs) were calculated following the standard methodology of the Global Burden of Disease (GBD) study. DALY is the sum of years of life lost due to premature mortality (YLL) and years lived with disability (YLD). All indicators were estimated by age and sex for each country.

YLL measures the disease-specific impact of mortality on the population’s health, taking into account the age of the deceased. YLL was computed by multiplying the number of deaths at each age by the corresponding standard life expectancy, using the GBD 2019 reference life table (21):


$$\mathrm{YLL}=\sum_{i}D_{i}\times {\mathrm{LE}}_{i}$$

Years lived with disability (YLD) was estimated by multiplying the prevalence of TB by the relevant disability weights (DW) and the average duration of disease. We used the GBD disability weights: 0.333 for TB without HIV and 0.408 for TB with HIV (22). TB with HIV was rare in our dataset and was not analyzed separately:


$$\mathrm{YLD}=\sum_{i}P_{i}\times {\mathrm{DW}}_{i}\times L$$

In estimating the YLD, we could distinguish between multidrug-resistant TB, extensively drug-resistant TB, and drug-susceptible TB. In both countries, the treatment duration for drug-susceptible TB 6 months and 9–24 months for drug-resistant forms, assuming a weighted average of 12 months. Therefore, we assumed these cases were exposed for 12 months of the year under consideration.

To compare the results between the two countries, we estimated the YLD, YLL, and DALY rates per 100,000 population and age-standardized them using the World Standard Population provided by GBD (21).

## 3 Results

### 3.1 Mortality and case fatality rates

According to our analysis based on official national data, all indicators are significantly higher in Mongolia than in Kazakhstan (for instance, MR for men is approximately 1.5 times higher).

An analysis of tuberculosis (TB) mortality rates from 2017 to 2021 showed clear differences between Kazakhstan and Mongolia in overall levels, trends over time, and patterns by age and gender (Table 1). In Kazakhstan, the TB mortality rate fell from 4.54 per 100,000 in 2017 to 3.95 in 2021 (a 13.0% decrease). In Mongolia, the drop was even greater — from 6.96 to 5.40 (a 22.4% decrease). Despite the decline in both countries, Mongolia consistently had higher mortality rates than Kazakhstan, by about 35–60% each year.

**Table 1:** TB mortality rate (per 100000) in Kazakhstan and Mongolia by sex and age group, 2017–2021.

		Kazakhstan					Mongolia				
		2017	2018	2019	2020	2021	2017	2018	2019	2020	2021
Male	0–14	0.20	0.04	0.00	0.04	0.07	0.41	0.20	0.19	0.37	0.18
	15–24	0.56	0.25	0.42	0.50	0.50	2.52	3.43	2.24	1.36	0.50
	25–34	3.93	3.05	3.11	3.85	1.85	9.34	4.92	5.84	7.04	1.85
	35–44	10.75	10.84	9.98	14.64	8.02	13.05	20.87	18.62	12.13	8.02
	45–54	14.49	13.58	13.89	15.17	11.65	25.74	27.44	35.44	26.55	11.65
	55–64	14.31	14.95	17.98	21.84	15.83	33.62	29.06	44.41	28.15	15.83
	65+	27.01	22.17	25.17	32.99	27.44	32.19	29.41	28.16	35.67	27.44
Female	0–14	0.00	0.00	0.04	0.00	0.04	0.22	0.21	0.40	0.39	0.19
	15–24	0.66	0.51	0.52	0.52	0.17	2.13	1.31	3.20	1.85	1.85
	25–34	1.79	1.40	1.09	2.26	0.99	4.11	2.81	4.40	1.86	3.41
	35–44	2.94	3.47	2.63	3.67	3.44	5.62	6.52	4.81	6.38	3.30
	45–54	2.64	4.19	3.74	4.01	3.08	4.39	8.70	8.13	6.34	5.70
	55–64	5.22	3.58	4.19	5.30	2.65	10.40	8.86	9.15	7.29	2.80
	65+	11.89	12.53	11.53	14.02	9.93	18.69	17.87	24.24	16.02	6.48
Male total		6.54	6.04	6.45	8.21	5.76	9.96	10.18	11.81	9.29	8.33
Female total		2.67	2.75	2.54	3.27	2.24	4.08	4.21	4.88	3.76	2.56
Total		4.54	4.35	4.44	5.67	3.95	6.96	7.16	8.30	6.49	5.40

MR, Mortality rate.

In all years and age groups, TB mortality was significantly higher in men than in women. In Kazakhstan, male mortality was on average twice as high as female mortality (e.g., in 2021: 5.21 vs. 2.64 per 100,000). In Mongolia, the gap was even larger — up to three or four times (e.g., in 2021: 8.33 vs. 2.56). The biggest difference was in the 65 + age group: in 2021, mortality among Mongolian men was 27.44, compared to 6.48 among women (4.2 times higher).

The highest mortality among men in both countries was found in the 65 + group. Among younger adults (15–34 years), Mongolia had consistently higher rates, especially in men (e.g., men aged 25–34 in 2021: 2.84 in Mongolia vs. 1.94 in Kazakhstan). Female mortality was lower in all age groups. In the 65 + group, a shift occurred: in 2017, mortality was higher in Mongolia (18.69 vs. 11.89), but by 2021 Kazakhstan had the higher rate (9.93 vs. 6.48).

In 2020, both countries experienced a peak in TB mortality, likely influenced by the COVID-19 pandemic. In Kazakhstan, the overall mortality rate increased from 4.44 in 2019 to 5.67 in 2020 (+27.7%), with the most notable gripe among older adults. Similarly, Mongolia recorded its highest rate over the five-year period — 8.30 in 2020, which was 16% higher than in the previous year. In 2021, both countries showed a decline in TB mortality compared to 2020: in Kazakhstan, the rate decreased by 30.3% (from 5.67 to 3.95), and in Mongolia — by 34.9% (from 8.30 to 5.40).

An analysis of tuberculosis (TB) case fatality rates (CFR) from 2017 to 2021 revealed notable differences between Kazakhstan and Mongolia (Table 2). In Kazakhstan, the overall CFR rose sharply from 4.96% in 2017 to 9.64% in 2020—an increase of 94.4%—before declining to 6.46% in 2021. In contrast, Mongolia experienced a more modest increase in CFR, from 4.36% in 2017 to 5.56% in 2021, representing a 27.5% rise. Throughout the entire period, CFRs in Kazakhstan remained consistently higher than in Mongolia, by approximately 25 to 70% each year.

**Table 2:** TB case fatality rates in Kazakhstan and Mongolia, 2017–2021, (in %).

		Kazakhstan					Mongolia				
		2017	2018	2019	2020	2021	2017	2018	2019	2020	2021
Male	0–14	2.36	0.64	0.00	0.83	1.64	0.96	0.60	0.48	0.94	0.67
	15–24	0.53	0.28	0.52	0.86	1.06	0.91	1.29	0.90	0.66	0.64
	25–34	2.75	2.49	2.73	4.88	2.59	4.22	2.43	2.60	3.48	2.88
	35–44	5.18	5.85	5.91	11.13	6.47	5.89	9.91	8.40	5.79	10.04
	45–54	7.27	7.24	7.77	11.14	7.76	9.94	11.79	12.46	10.98	10.62
	55–64	7.85	9.17	10.34	15.94	10.89	13.10	11.50	18.56	11.29	15.37
	65+	15.93	15.17	17.01	27.42	21.39	13.13	10.89	11.09	16.57	22.85
Female	0–14	0.00	0.00	0.49	0.00	0.74	0.39	0.67	1.02	0.88	0.61
	15–24	0.68	0.59	0.64	0.94	0.31	0.90	0.63	1.21	0.96	1.35
	25–34	1.67	1.49	1.15	3.41	1.59	1.91	1.37	2.17	0.95	2.57
	35–44	1.67	4.41	3.51	6.10	5.87	4.63	5.16	3.97	5.03	3.61
	45–54	3.85	6.94	6.48	8.41	6.76	3.57	7.14	6.81	5.09	6.92
	55-64	8.54	6.17	7.02	11.52	5.93	8.14	7.17	7.21	5.88	3.30
	65+	12.11	13.28	12.69	19.52	13.41	10.73	11.15	17.64	10.44	7.46
Male total		5.56	5.86	6.57	11.05	7.88	5.51	6.11	6.77	5.93	7.44
Female total		3.96	4.54	4.32	7.41	5.25	2.92	3.40	3.75	3.09	3.09
Total		4.96	5.35	5.69	9.64	6.87	4.36	4.94	5.46	4.67	5.56

CFR, Case-fatality rate.

Case fatality rates were consistently higher among men across all age groups. For example, in Kazakhstan in 2020, the CFR among men reached 11.05%, compared to 7.41% among women (1.5 times higher). In Mongolia, the gender gap was even more pronounced in 2021, with a CFR of 7.44% in men versus 3.09% in women (2.4 times higher).

The highest CFRs were observed in the 65 + age group. In Kazakhstan, the rate peaked in 2020 at 31.64% for men and 19.52% for women. In comparison, Mongolia reported lower values in the same year: 11.25% among men and 10.44% among women.

In 2020, during the COVID-19 pandemic, a noticeable increase in case fatality rates was observed in both countries. In Kazakhstan, the total CFR rose by 69.4% compared to 2019 (from 5.69 to 9.64%), while in Mongolia the increase was 16.9% (from 4.67 to 5.46%). The most significant contribution to this growth came from older age groups, particularly men aged 65 and over.

Based on the reported figures from both countries, MR and CFR were lowest in the younger age groups (0–14 and 15–24), with relatively small gender differences. In Mongolia, the MR for male children aged 0–14 decreased slightly from 0.41 in 2017 to 0.18 in 2021, while the rates for female children remained stable around 0.22. Among adults aged 25–44, both countries reported higher MR and CFR for men. In Kazakhstan, the MR for males aged 35–44 peaked at 14.64 in 2020, while in Mongolia, male MR in this group reached 20.87 in 2018 before showing a declining trend. Among older adults (45 + years), male MR consistently exceeded female MR in both countries. In Kazakhstan, MR for males aged 65 + peaked at 32.99 in 2020, while in Mongolia, the same group recorded its highest MR at 35.67 in 2020 before declining to 27.44 in 2021.

### 3.2 Disease burden of TB

#### 3.2.1 Overall trends in TB burden (YLL, YLD, and DALY) in Kazakhstan and Mongolia

**Figure 1:**
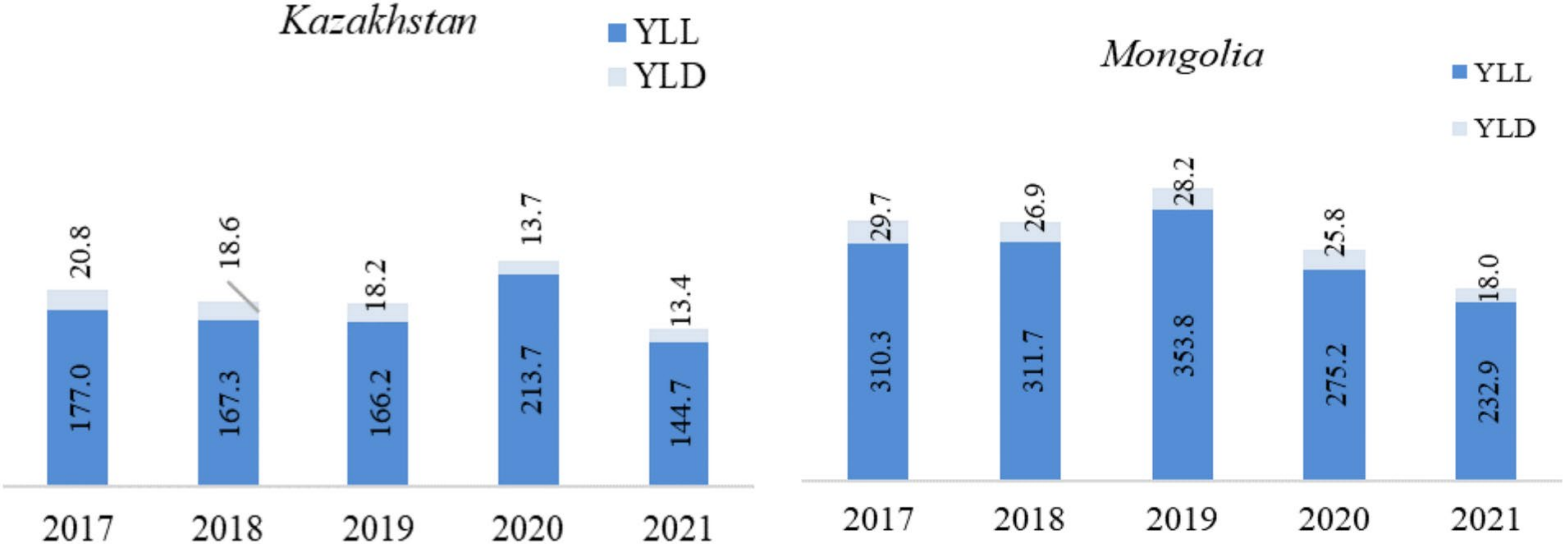
Trends in YLL, YLD, and DALY (per 100,000 population) in Kazakhstan and Mongolia (2017–2021). *YLL, Years of life lost from mortality. **YLD, Years of healthy life lost due to disability. ***DALY, Disability-adjusted life years.

Examining the burden of TB in Kazakhstan and Mongolia for the years 2017 to 2021, based on reported figures, a substantial reduction in both YLL and YLD was observed (Figure 1). In Kazakhstan, the YLL rate declined from 177.0 in 2017 to 144.7 in 2021, reducing TB-related mortality. The YLD rate decreased from 20.8 in 2017 to 13.4 in 2021. There was a significant increase in 2020 when the YLL rose to 213.7. In 2020, the COVID-19 pandemic led to a 38.9% increase in male YLL in Kazakhstan, while Mongolia exhibited a peak YLL rate of 353.8 in 2019, reflecting delayed access to care. In 2017, the total DALYs were 197.8 (177.0 YLL and 20.8 YLD). By 2021, this had decreased to 158.1 (144.7 YLL and 13.4 YLD), an overall DALY reduction of about 20.1%.

In Mongolia, the YLL rate peaked at 353.8 in 2019 but decreased by 34.2% to 232.9 in 2021, indicating a reduction in TB-related mortality. The YLD rate also showed improvement, falling from 29.7 in 2017 to 18.0 in 2021, representing a 39.4% reduction in disability rates. The combined DALY rate was highest in 2019 at 382 and decreased by 34.3% to 250.9 in 2021.

Mongolia experienced a significant 24.9% reduction in YLL, from 310.3 in 2017 to 232.9 in 2021, and a 39.4% reduction in YLD, indicating an overall improvement in mortality and disability rates. Mongolia had consistently higher YLL and YLD rates than Kazakhstan, indicating a higher disease burden. However, both countries showed positive development in reducing TB-related mortality and disability over the period (Figure 1).

#### 3.2.2 Gender differences in the TB burden

**Figure 2:**
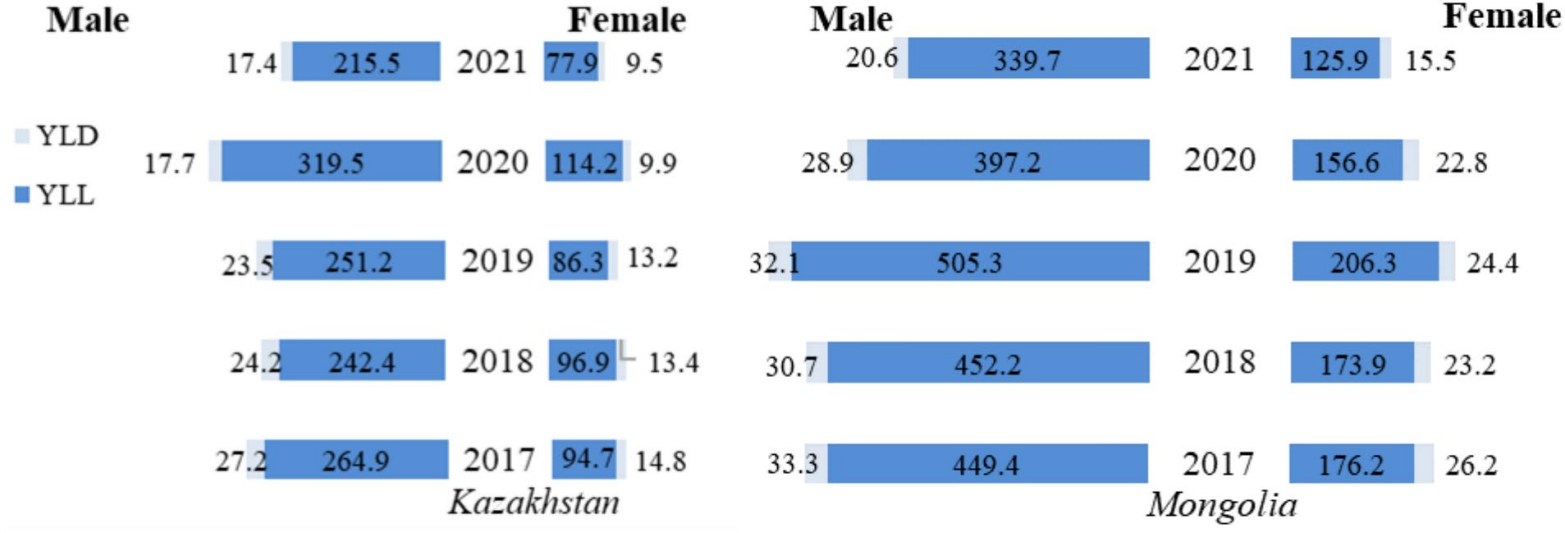
Gender differences in tuberculosis YLL, YLD, and DALY rates (per 100,000 population) in Kazakhstan and Mongolia, 2017–2021.

Comparing DALY rates by sex, the burden of TB was substantially higher in men than in women (Figure 2). In particular, between 2017 and 2021 in Kazakhstan, based on reported figures, YLL for men decreased from 264.9 to 215.5, a reduction of 18.6%, and for women from 94.7 to 77.9, a reduction of 17.7%. YLL rates peaked in 2020 for both men and women, reaching a maximum of 319.5 and 114.2, respectively. YLD rates fluctuated less, falling from 27.2 to 24.2 for men and 14.8 to 9.5 for women.

A similar overall decline was observed in Mongolia. YLL rates for men fell from 449.4 in 2017 to 339.7 in 2021, a decrease of 24.4 percent, while for women, they fell from 176.2 to 125.9, a decrease of 28.6 percent. However, the highest YLL rates were observed in 2019 (not in 2020 as in Kazakhstan) where in men it reached the level of 505.3, and in women the peak was at 206.3. YLD rates also declined for the observed period, with men experiencing a decrease from 33.3 in 2017 to 20.6 in 2021 and women from 26.2 to 15.5.

Kazakhstan and Mongolia experienced notable reductions in TB burden between 2017 and 2021, though the patterns varied. In Kazakhstan, DALY rates for men decreased by 20.27% (from 292.1 in 2017 to 232.9 in 2021), while for women, the reduction was 20.18% (from 109.5 in 2017 to 87.4 in 2021). Conversely, Mongolia showed even more significant reductions, with DALY rates falling by 25.36% for men (from 482.7 in 2017 to 360.3 in 2021) and 30.14% for women (from 202.4 in 2017 to 141.4 in 2021). Over the period considered, these figures show more significant progress in reducing the TB burden in Mongolia, which started from a much higher level than in Kazakhstan. However, while both countries experienced fluctuations, Kazakhstan maintained lower mortality and disability rates than Mongolia.

#### 3.2.3 Age-specific patterns in the TB burden: comparison between 2017 and 2021

**Figure 3:**
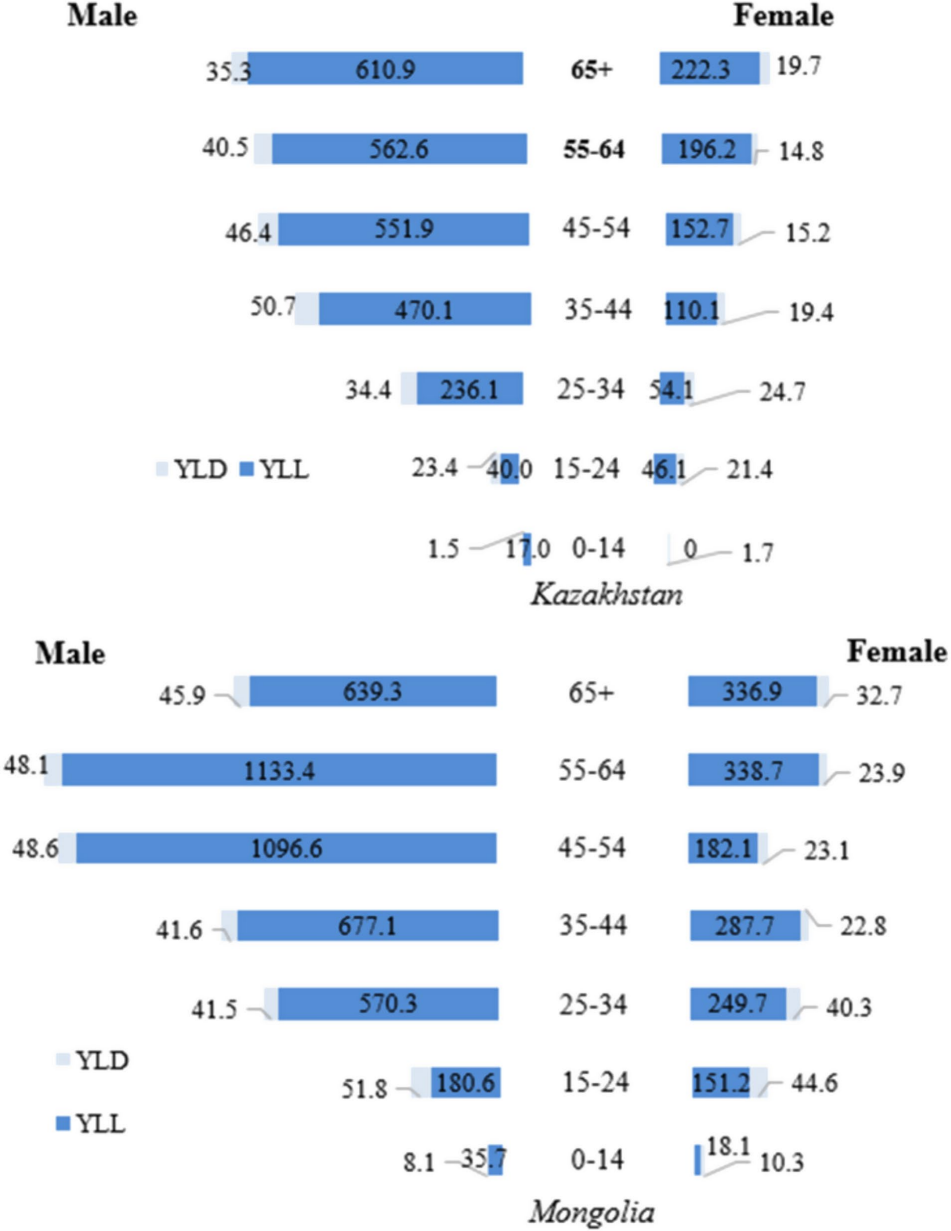
Gender differences in tuberculosis YLL, YLD, and DALY rates (per 100,000 population) by age group in Kazakhstan and Mongolia, 2017.

In 2017 (Figure 3), the main contributor to the high TB DALY rates in both Mongolia and Kazakhstan was the YLL metric, which reflects premature mortality. For men aged 45–54, the YLL rate in Mongolia was 1096.6, more than double the rate in Kazakhstan (551.9). In the 55–64 age group, the YLL rate in Mongolia was about twice as high (1133.4 compared with 562.6 in Kazakhstan). For women, the largest difference was in the 65 + age group, where Mongolia’s YLL rate (336.9) was 51.6% higher than Kazakhstan’s (222.3). Among younger cohorts (0–14 and 15–24 years), the YLL rate was slightly higher in Mongolia, with a notable difference observed among males aged 15–24 (180.6 vs. 23.4 in Kazakhstan). YLD rates remained consistently low compared to the YLL rate. They had minimal impact on total DALYs, underscoring that premature mortality, particularly among older men, was the dominant factor driving high DALY rates (Figure 3). The sex differences persist in all age groups, both in Kazakhstan and Mongolia. YLL and YLD rates are predominantly higher in men compared to women.

**Figure 4:**
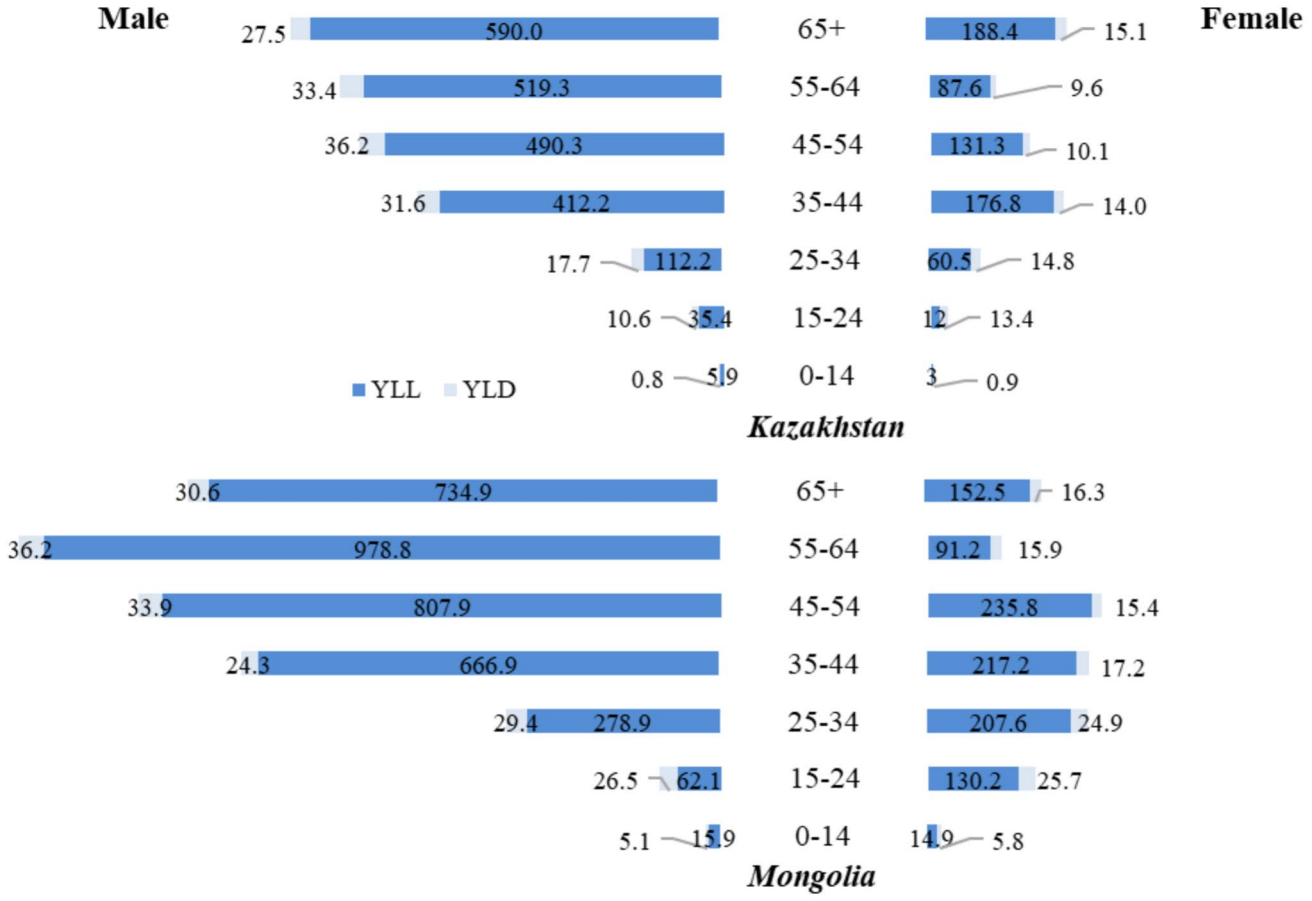
Age and gender differences in tuberculosis YLL and YLD rates (per 100,000 population), 2021.

In 2021 (Figure 4), trends in DALYs from TB in Mongolia and Kazakhstan were again primarily influenced by the number of YLL. In Mongolia, the YLL rate for men 65 years and older was 734.9, approximately 1.25 times higher than in Kazakhstan, where the rate was 590.0. Among women 65 years and older, the YLL rate in Kazakhstan (188.4) was approximately 1.24 times higher than in Mongolia (152.5), highlighting a smaller disparity than men in the same age group. For men aged 55–64 years, the YLL rate in Mongolia (978.8) was approximately 1.88 times higher than in Kazakhstan (519.3), reflecting a significant disparity in TB-related mortality between the two countries.

The disparity was also notable in younger age groups. Among men aged 15–24, the YLL rate in Mongolia (62.1) was approximately 1.76 times higher than in Kazakhstan (35.4). Among women in the same age group, the YLL rate in Mongolia (130.2) was about 10 times higher than in Kazakhstan (12), highlighting significant differences in TB-related mortality. In both countries, YLD rates remained relatively low. They had little impact on the overall burden of TB, with the highest YLD rates occurring among men aged 45–54 years in Kazakhstan at 36.2 (Figure 4).

During the observed period, the YLD rate in Kazakhstan constituted approximately of 63% for drug-susceptible TB and 37% for drug-resistant TB. In contrast, for Mongolia it was estimated an 80% YLD rate for drug-susceptible TB and 20% for drug-resistant TB (data not shown).

The disease burden of TB was concentrated in the working-age population. DALYs, YLL, and YLD were higher in males than females in all years.

## 4 Discussion

According to officially reported data, the overall DALY of TB decreased for both countries in 2021 compared to 2017. Kazakhstan experienced a reduction from 197.8 to 158.1 (20.07%), while Mongolia’s DALYs decreased from 340.0 in 2017 to 250.9 in 2021 (26.21%). However, between 2017 and 2020, Kazakhstan’s TB DALY rate fluctuated, initially decreasing from 197.8 in 2017 to 185.9 in 2018, followed by a sharp increase to 227.3 in 2020. This pattern highlights a temporary decrease in the burden of TB, which was subsequently offset by a significant increase, reflecting a probable impact of the COVID-19 pandemic. The burden of disease due to TB in the Republic of Kazakhstan, as measured by the DALY indicator, stands at 16449.54 per 100,000 population (falling within the range of 15243 to 17749 DALY per 100,000 population) (23). This overall decline in the disease burden suggests progress in reducing both premature death and disability due to TB. The most significant changes occurred between 2019 and 2021, with substantial decreases in mortality and disability.

Between 2017 and 2021, based on reported figures, there were notable decreases in both YLL and YLD rates, reflecting a reduction in the TB burden in Kazakhstan and Mongolia. In Kazakhstan, the YLL rate fell by 18.2%, reflecting a modest reduction in TB-related mortality. In comparison, the YLD rate decreased by 35.6%, reflecting improved patients’ quality of life. Although YLL decreased by 24.9% in Mongolia and YLD by 39.4%, the overall disease burden remained significantly higher than in Kazakhstan. Premature mortality, particularly among older men, remained the main driver of high DALY rates in both countries. YLD rates remained consistently low and did not significantly impact the overall DALY figures. These results highlight positive trends in reducing TB mortality and disability in both countries.

A study conducted from 2014 to 2019 revealed a consistent decline in TB incidence, decreasing from 227.0 to 69.1 per 100000 population in Kazakhstan. However, the mortality rate observed in the study persistently elevated (24). The findings by Yerbolat S. et al. demonstrated a decline in TB incidence alongside a rise in overall mortality among TB patients over the 5 years under study. In contrast, our study found a slight decrease over the period 2017 to 2019 (24). Research carried out in Mongolia between 2015 and 2019 indicated a decline in the number of registered TB cases from 2015 to 2018, followed by an increase in 2019, with 133 new cases and relapses per 100,000 population (25). Our findings align with this observation. The WHO has reported a reduction in the incidence of TB in Kazakhstan since 2000 (26, 27). Favorable trends in TB epidemiology have also been documented in neighboring countries, including Kyrgyzstan and Uzbekistan. For instance, Kozhoyarova et al. identified a gradual decline in TB mortality in Kyrgyzstan from 2007 to 2017 (28). Safayev and colleagues documented a notable decrease in reported TB cases in Uzbekistan over the past two decades (29).

In 2003, the Government of Mongolia initiated a collaboration with the Global Fund to Fight AIDS, TB, and Malaria. Over the past two decades, eight projects have been successfully implemented with financial support from the Fund, leading to significant improvements in TB treatment and diagnosis, decentralization of TB care, and increased access to services. Our study confirmed a 34.3% reduction in DALYs in Mongolia between 2017 and 2021, demonstrating the potential impact of these initiatives. This collaboration and funding may have contributed significantly to reducing the TB burden in the country (30).

To address the burden of TB in Kazakhstan, a Memorandum of Understanding was signed between the Government of the Republic of Kazakhstan and the Global Fund to Fight AIDS, TB, and Malaria (31). This initiative has implemented comprehensive measures to reform the TB control system, strengthen the management of drug-resistant TB, expand access to diagnostic and treatment services, and address the specific needs of vulnerable populations. The results of our study confirm the potential impact of these interventions, indicating a reduction in the burden of TB (DALY) to 158.1 per 100,000 population in 2021, a reduction of 20.07% compared to 2017 (197.8 per 100,000) (32).

In 2017, cross-border cooperation to combat multidrug-resistant TB (MDR-TB) was established in Kazakhstan to reduce the incidence of the disease among migrant workers. In the same year, the National Partnership “STOP TUBERCULOSIS” was established, with the main goals of ensuring access to effective diagnosis and access to TB healthcare services for all patients, interrupting the transmission of the infection, reducing its social and economic impact, and developing and implementing new tools and strategies to combat the disease. These efforts contributed to a reduction in TB mortality rates in the following years. Between 2014 and 2020, a national TB control program was developed, with a target to reduce mortality to 5.8 per 100,000 (down from 8.0 per 100,000 in 2012) by 2020. Our results revealed a reduction in mortality to 4.4 per 100,000 by 2019. However, a significant increase in 2020 was found, with the YLL rate rising to 213.7, possibly due to external factors such as the COVID-19 pandemic affecting access to healthcare. Other studies have shown that the COVID-19 pandemic led to an increase in mortality in 2020 with 5.67 per 100,000 in that year (33).

To achieve WHO global TB elimination goals by 2030, technological breakthroughs must be implemented by 2025 (34). Policy efforts should focus on cross-border TB monitoring, community-based care, and improved adherence strategies to reduce TB-related mortality (35). Significant progress has been made in developing new technical tools. In 2013, the WHO approved seven diagnostic tools, notably including the Xpert MTB/RIF, a sensitive molecular test for rapid TB diagnosis and rifampicin resistance detection. By mid-2013, 1,402 GeneXpert devices and 3.2 million cartridges had been purchased by 88 of the 145 low- and middle-income countries eligible for preferential pricing (36). In Kazakhstan, Xpert MTB/RIF Ultra devices, introduced in 2018, has proven effective for the rapid and accurate diagnosis of drug-resistant TB, ensuring timely and appropriate treatment (37). Similarly, in Mongolia, the Xpert test facilitated the early detection of TB, including drug-resistant forms, enabling prompt treatment according to the recommended regimens (38). Furthermore, the introduction of GeneXpert has helped to expand coverage in rural areas of the country (39). In addition, various laboratory tests, together with the use of the latest TB diagnostic technologies and treatment regimens, contribute to timely and effective disease management. Based on our results, we hypothesize that improving patients’ quality of life is closely linked to early diagnosis, timely treatment and appropriate treatment of DR-TB, as these factors are critical in reducing disease progression and improving patient outcomes; the YLD rate also decreased slightly in both countries.

Practical laboratory and information systems for TB control have been developed in many countries over the past decades (40). In Kazakhstan, the Ministry of Health oversees and finances TB prevention and treatment, as well as oversees regional primary healthcare organizations and TB services. The primary preventive measures, which extend beyond WHO recommendations, include administering the Bacillus Calmette-Guerin vaccine to infants and children, conducting annual TB skin tests for school-age children, performing chest X-rays for adults, and monitoring potential TB contacts (41, 42). TB patients with a negative smear or culture can receive outpatient treatment (43, 44). Meruert D. and co-authors assert that the outpatient TB treatment model produces results comparable to inpatient treatment. These programs help to reduce the stigma of TB and provide the necessary community support (45).

Service interruptions caused by the COVID-19 pandemic have potentially led to diagnostic and treatment delays (46). In many countries, human, financial, and other resources have been reallocated from TB control to the COVID-19 response. This shift has also had a negative impact on data collection and reporting systems. One of the key global challenges is funding. In 2020, only $6.5 billion was allocated for TB prevention, diagnosis, treatment, and care, only half of the $13 billion target set by world leaders in the UN Political Declaration on Tuberculosis (34).

In Kazakhstan, the decline in YLL for men was 18.6%, compared to 17.7% for women. In contrast, Mongolia had higher YLL in both sexes, with substantial disparities, especially in older age groups, indicating a more pronounced disease burden among men. This could reflect the sex-specific social contacts pattern, where men are usually more exposed to TB infections (8). Other studies report a higher survival rate among women with TB compared to male patients. For instance, Maissa Ben Jmaa et al. demonstrated that women with TB exhibited a higher likelihood of recovery compared to men (47). Furthermore, Chidambaram et al. reported in their retrospective cohort study that men show a higher mortality rate from all causes in comparison to women (48). The age-related gender disparities in TB incidence rates observed across multiple countries underscore the significance of incorporating sex as a biological variable when assessing TB risk factors (8).

Our study further corroborated the gender disparity in survival, highlighting that women are less likely to succumb to TB-related mortality. According to our analysis, the burden of TB remained higher among older men in Mongolia, with YLL for men aged 65 + significantly surpassing that in Kazakhstan. Notably, in 2021, Mongolia’s YLL for this age group was1.25 times higher than Kazakhstan’s. The study also supported the finding that age is the third most common risk factor for TB, particularly affecting individuals aged 65 and older. The immune system weakens with age, reducing the body’s resistance and making older adults more susceptible to various diseases (49). Higher YLL rates in Mongolia may reflect rural–urban healthcare disparities, limited diagnostic capacity, and delayed case detection, particularly in older adults (50). Another explanation for the consistently higher TB rates in older ages is considered to be the higher prevalence of latent TB infection. Older age cohorts have been exposed in times where TB incidence were much higher and had more opportunities to get infected (51). A possible explanation for the higher YLL observed in Mongolia than in Kazakhstan, could be the fact that a high percentage of TB patients in Mongolia lives in Ulan Bator, which is the city with highest air pollution worldwide. People suffer from lung damages because of this, and this might lead to higher mortality especially for TB patients compared to KAZ. Furthermore, the impact of air pollution and the caused lung diseases increases by age. Additionally, older adults are more likely to have other chronic conditions, which further increases the risk of reactivating TB (52, 53). In our study, we also confirmed that the YLL rate is higher among men over 65 (2017, Kazakhstan, 610.92 per 100,000).

In Kazakhstan, MR in males peaked at 32.99% in 2020 among men aged 65+, while in Mongolia the peak was in 2019 in men at age group 55 – 64 (44.41). This highlights the need for further and more targeted public health strategies to reduce the TB burden, particularly among the more vulnerable older populations.

The comparison of mortality rates between the WHO and national databases has yielded discernible disparities. Since 2017, the national database has shown significantly higher mortality rates than the WHO database. The difference was high in 2020, reaching 62%, with Kazakhstan’s database exhibiting much higher estimations. Examining mortality rates across age groups in both databases has unveiled noteworthy distinctions. Before 2017, the national database consistently recorded higher rates, particularly among age groups surpassing 60.

The World Health Organization’s 2017 report a significant decline in adjusted TB mortality from 2005 to 2015. However, the authors, including Yesbolat S., observed that overall mortality nearly doubled between 2014 and 2019 (24). This increase in mortality during this time period may be related to excessive hospitalization of TB patients in Kazakhstan, despite WHO recommendations for outpatient treatment to reduce the risk of nosocomial transmission of drug-resistant strains (24, 45). Furthermore, the increased mortality among TB patients may be due to comorbidities. Our study corroborates these findings and shows a significant increase in YLL rates in Kazakhstan, particularly in 2020 and 2021. In 2020, the YLL rate for males increased by 38.9%, from 264.9 in 2017 to 367.7 in 2020. For women, the YLL rate increased by 40.9%, from 94.7 in 2017 to 133.3 in 2020. These increases reflect the worsening burden of TB, particularly during the pandemic. These results highlight that health care systems should be prepared and able to ensure continuation of TB prevention (e.g., infection control and contact tracing) and care even in times of crisis such as a pandemic. Lin and co-authors argue that non-communicable comorbidities, particularly liver cirrhosis, HIV infection, and multidrug-resistant TB, are significant risk factors for death among TB patients (54). The WHO reports that the incidence of multidrug-resistant TB (MDR-TB) remains consistently high in Kazakhstan. For example, MDR to rifampicin (MDR/RR) accounts for about 27% of new TB cases and 44% of cases on TB treatment (55).

Mongolia has demonstrated complex trends in TB mortality over the past two decades (56). However, as noted by authors such as Tsolmon Boldoo, the number and rate of TB notifications decreased during 2015–2018 but rose again in 2019, despite increased screening efforts (25). Our study confirmed this development, with the YLL rate peaking at 353.8 in 2019 and decreasing by 34.2% in 2021. Similarly, the combined DALY rate was highest in 2019 at 382, declining by 34.3% to 250.9 in 2021. The increased TB notifications and bacteriological confirmation in 2019 was attributed to expanded X-ray and Xpert testing and improved specimen transportation systems (25). Despite these advancements, the WHO estimated that Mongolia’s incidence of RR/MDR-TB remains among the highest in the Western Pacific Region (57). On the other hand, TB diagnoses and DR-TB numbers may have increased because of diagnostic improvements and roll-out of rapid molecular tests. Gurjav U. et al. highlight a concerning trend of MDR-TB transmission, driven primarily by the spread of Beijing lineage strains resistant to all first-line drugs (58). In 2019, 92% of RR/MDR-TB cases were enrolled in second-line treatment, reflecting an improvement from 85% in 2015 (25).

We observed a slight discrepancy when comparing our results with those from other databases (e.g., WHO, GBD). For example, YLD rates in Kazakhstan since 2017 for males, females, and both sexes were nearly identical between GBD and Kazakhstan data, with minimal differences of −0.4, −9.1%, and −3.6%, respectively. Over the years, these differences fluctuated significantly, with the enormous gap occurring in 2020 when Kazakhstan’s data showed a decrease of 32.4% for males and 39.0% for females compared to the GBD data. By 2021, the differences remained significant, with Kazakhstan showing a reduction of −26.7% for males, −43.0% for females, and −32.7% for both sexes. In Mongolia, the same indicator was lower than GBD data for males, females, and both sexes, with differences of −30.2, 5.6%, and −14.4%, respectively. These differences fluctuated over the years, with the most significant discrepancy observed in 2021. In that year, the Mongolian data showed a significant decrease of −65.3% for males, −24.4% for females, and −47.5% for both sexes compared to the GBD data (23).

YLL rates since 2017 in Kazakhstan were substantially higher than in GBD data, with an increase of 14.5% for males, 12.0% for females, and 13.8% for both genders. Between 2018 and 2019, the differences remained relatively stable, fluctuating around 11.3% in 2018 and 16.6% in 2019. However, the largest differences occur in 2020 and 2021, with Kazakhstan showing much more significant increases, especially in 2020, with differences of 38.9% for men, 40.9% for women, and 39.4% for both sexes. By 2021, the gap will be reduced to 9.8% for men and 14.4% for women, reflecting a clear upward trend in Kazakhstan compared to GBD. In Mongolia, the same indicator was consistently lower than the GBD data in all years. In 2017, the differences were significant at −170.2% for males, −112.1% for females, and −154.4% for both sexes. This trend continued, with the most notable differences observed in 2021, when the gaps reached −190.0% for males, −110.5% for females, and −166.5% for both genders (23).

According to the 2019 GBD study results, TB has consistently ranked as the third most prevalent disease in Central Asia over the past three decades. In 2019, the TB burden of disease, measured in DALYs, was 2,032.23 per 100,000 population. There is a consistent pattern of gradual decline in TB incidence across Central Asian countries. However, despite this trend, the burden of the disease remains a significant challenge. In 2019, Mongolia emerged as the country with the highest burden of TB, with DALY rates of 557.51 per 100,000 population.

### 4.1 Strength and limitation

This study is the first of its kind in Kazakhstan and Mongolia. It comprehensively analyses TB morbidity and mortality trends over recent years and their impact on DALY, YLL, and YLD indicators.

The data were obtained from registration databases, which implies potential inaccuracies due to data entry errors and procedures associated with collecting and registering information. Data accuracy was ensured through cross-validation with national TB registries. However, underreporting during the COVID-19 pandemic remains a potential limitation. COVID-19 disrupted TB care pathways, leading to delayed diagnosis and increased mortality, which may overestimate TB burden in 2020. In addition, due to the unavailability of complete and comparable subnational data, we were unable to assess regional differences in the TB burden in Kazakhstan and Mongolia. Comparisons with GBD estimates for Kazakhstan and Mongolia reveal significant differences between national data and estimates from international comparative analyses. On the one hand, national data are likely to be underestimated. On the other hand, short-term developments during the COVID-19 pandemic or the post-epidemic decline in the TB burden are not reflected in the international data to the same extent as in the more simulation-based GBD study. This is a preliminary finding and due to the short time series, should be validated with future data.

## 5 Conclusion

Our study, using national health data from 2017 to 2021, provides important insights into the burden of TB in Kazakhstan and Mongolia. The results show an overall decline in TB morbidity and mortality. Examining trends in YLL, YLD, and the composite measure of DALY over the period of 5 years revealed that YLL contributes significantly more to the DALY than YLD. In addition, we identified age and gender discrepancies: the predominant TB burden was observed in the working-age male population. The DALY, YLL, and YLD metrics were consistently higher in males than in females throughout the study.

Our findings provide a more nuanced picture of the structure of the TB burden, thereby guiding the development of timely and effective TB intervention strategies and providing essential data for epidemiological and health economic research. This study highlights significant TB morbidity and mortality trends in Kazakhstan and Mongolia, revealing discrepancies between national data and international estimates. It highlights the need for improved national data accuracy, further analysis of differences in data sources, and the inclusion of long-term time series for more robust results. Sustained reductions in TB burden require investment in diagnostics, treatment equity, and cross-border collaboration to address regional disparities. In this context, these preliminary findings should be validated with future data to ensure comprehensive monitoring and better health outcomes.

## Data Availability

The raw data supporting the conclusions of this article are available from the corresponding author, upon reasonable request.
