# Correction: Trends and disparities in tuberculosis burden in Kazakhstan and Mongolia (2017–2021): a comparative analysis using GBD metrics

**DOI:** 10.3389/fpubh.2025.1649874

**Published:** 2025-07-15

**Authors:** Oyunzul Amartsengel, Malika Idayat, Alexander Rommel, Natalya Glushkova, Kairat Davletov, Malik Adenov, Naranzul Dambaa, Lkhagvasuren Khorolsuren, Elena von der Lippe

**Affiliations:** ^1^Department of Health Policy, Mongolian National University of Medical Sciences, Ulaanbaatar, Mongolia; ^2^Department of Medicine and Public Health, Al-Farabi Kazakh National University, Almaty, Kazakhstan; ^3^Robert Koch Institute, Berlin, Germany; ^4^Science and Technology Park, Asfendiyarov Kazakh National Medical University, Almaty, Kazakhstan; ^5^Research Institute of Phthisiopulmonology, Almaty, Kazakhstan; ^6^National Center for Communicable Disease, Ulaanbaatar, Mongolia

**Keywords:** disability-adjusted life years, years of life lost, years lived with disability, TB, Central Asia

Author Lkhagvasuren Khorolsuren was erroneously assigned to affiliation 6 “National Center for Communicable Disease, Ulaanbaatar, Mongolia”. This affiliation has now been removed for author Lkhagvasuren Khorolsuren. Instead, they are assigned to affiliation 1 “Department of Health Policy, Mongolian National University of Medical Sciences, Ulaanbaatar, Mongolia.”

Author Elena von der Lippe was erroneously spelled as Elena Von Der Lippe, with capitalization incorrectly applied.

The original version of this article has been updated.

